# Supplementary material for: Synthesizing a ν=2/3 fractional quantum Hall effect edge state from counter-propagating ν=1 and ν=1/3 states
Source: Nat Commun. 2019 Apr 23;10:1920. doi: 10.1038/s41467-019-09920-5 (PMC6478935; doi:10.1038/s41467-019-09920-5)
Supplement: Supplementary file 1 — Supplementary Information [file 41467_2019_9920_MOESM1_ESM.pdf]

Synthesizing a ν=2/3 fractional quantum Hall effect edge state from counter-propagating ν=1 and ν=1/3 States

Yonatan Cohen et al.

Supplementary Note 1 – Double-quantum-well (DQW) heterostructure

Supplementary figure 1 is a schematic illustration of the MBE growth of the DQW heterostructure used to implement the two-subband (SB) system. The quantum-well structure consists of a 40 nm thick GaAs layer, cladded by AlGaAs layers on the top and bottom. A thin AlAs layer, is inserted in the center of the 40 nm quantum well, forming a potential barrier in the center of the QW. The density of SB1 is mostly located in the lower side of the well while most of the density of SB2 is located in the upper side of the well (see Ref. 20). The thickness of this AlAs used in this work are 0.7 nm and 1.5 nm, which affects the interaction between the electrons in the two subbands.

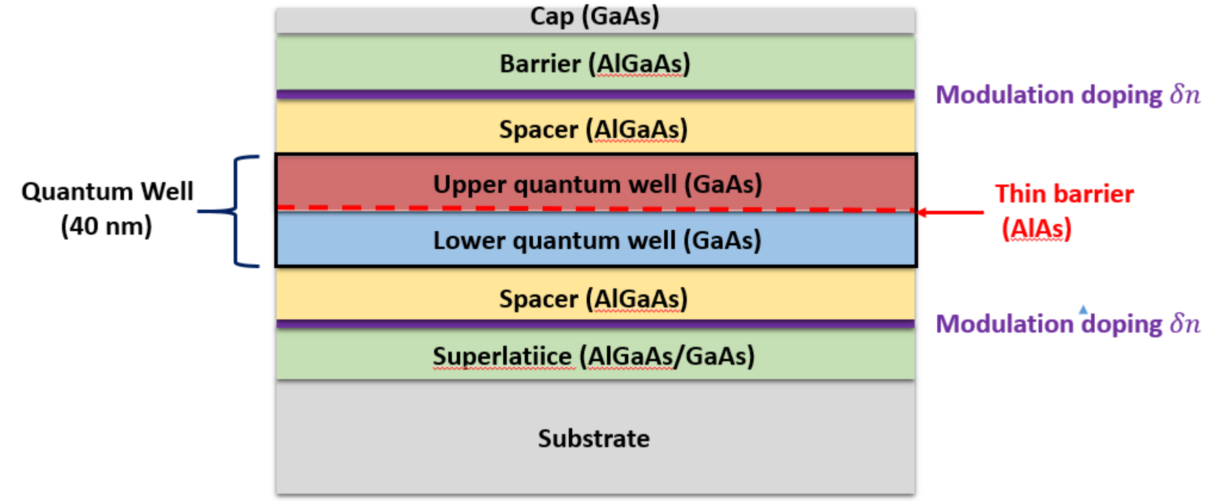

Supplementary Figure 1. A schematic diagram of growth sequence of the double-quantum-well (DQW) heterostructure. A thin AlAs layer is grown in the center of the 40 nm wide GaAs QW to separate the densities of the two subbands (SBs). The lower and upper GaAs quantum wells are colored in blue and red, respectively. Different thickness of AlAs layers (0.7 nm, 1.5 nm and 3 nm) are used in this work.

Supplementary Note 2 – R_{xx} measurement of a DQW with 1.5 nm AlAs barrier

Supplementary figure 2 shows the fan diagram of the longitudinal resistance R_{xx} as a function of magnetic field and gate voltage (used to tune the carrier density), measured in a DQW with 1.5 nm thick AlAs barrier. The dark blue regions of vanishing R_{xx} represent the quantum Hall phases at both integer and fractional filling factors, whose filling factors are labeled. In contrast to the 2DEG in DQW with 0.7 nm thick AlAs barrier (described in the main manuscript), the gap, separating (2,0) and (1,1), disappears due to weak inter-mode tunneling.

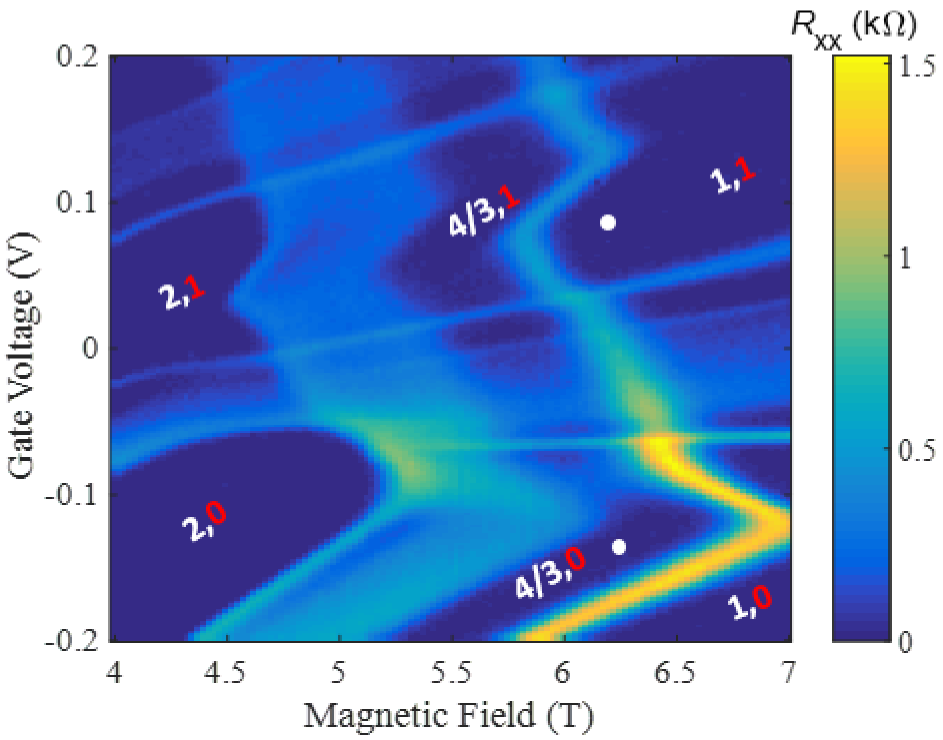

Supplementary Figure 2. The longitudinal resistance, R_{xx}, as a function of magnetic field and gate voltage. This fan diagram was measured in a DQW with 1.5 nm thick AlAs barrier.

Supplementary Note 3 – Bias dependence of the conductance

The non-linear differential conductance, at B=6.45 T, for a few propagation lengths, is shown in supplementary figure 3a. At short distances (L=6 μm & 15 μm), with a conductance at zero-bias being $G_{2T}=4e^2/3h$. With increased DC bias the conductance initially decreased sharply (up to ~150 μV and less), followed by a slower decrease at higher DC bias. In contrast, for the longest distance (L=150 μm), with a zero-bias conductance $G_{2T}=2e^2/3h$, the conductance smoothly increased with bias. In between (L=38 μm & 68 μm), the conductance decreased abruptly to $G_{2T}=2e^2/3h$ (at a range V_{DC}~35-60 μV), followed by a soft increase with increasing DC bias.

Supplementary figure 3b shows the differential conductance as a function of the DC bias for several magnetic fields at a propagation length of L=38 μm. At higher B (weak inter-mode coupling), a similar

zero-bias conductance peak appeared. At lower *B*; however, the conductance, G_{2T}~$2e^2/3h$, was independent on bias around zero bias; but experienced a rather steep increase as the bias increased. The critical bias, where the increase in the conductance took place, the conductance increased gradually with lowering the magnetic field. It seems that either the magnetic field or the biasing voltage, both suppress the inter-mode coupling, and thus increase the conductance. As the spin orientation is different for these two modes, such threshold behavior might be related to the energy scale determined by spin-flip. Yet, the interpretation of the behavior of the non-linear differential conductance is not trivial, and requires more studies.

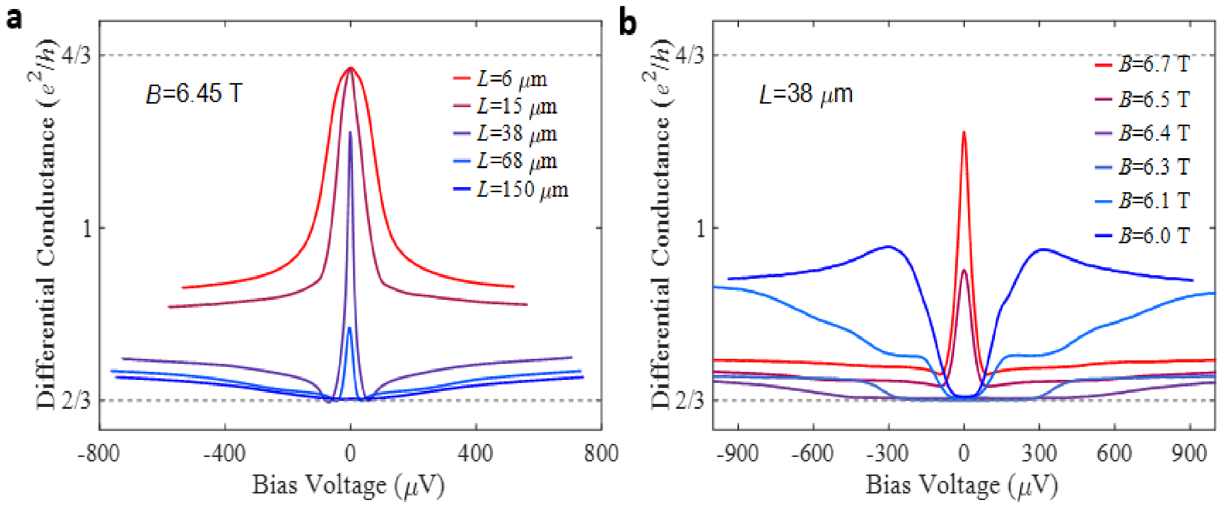

Supplementary Figure 3. The effect of an applied DC bias on the two-terminal differential conductance of the two counter-propagating *v*=1 and *v*=1/3 modes. **a.** At *B*=6.45 T, conductance versus bias voltages for different propagation lengths. **b.** At *L*=38 μm, conductance as a function of DC bias voltage for a few magnetic fields.

Supplementary Note 4 – Upstream and downstream noise at filling factor *v*=1

Supplementary figure 4 shows the measured excess upstream and downstream noise of a mode of *v*=1, formed at the interface of the upper region at (1,1) and center region at (1,0) (supplementary figure 4). This indicates that the appearance of upstream noise at filling factor *v*=2/3 comes from the expected upstream noise.

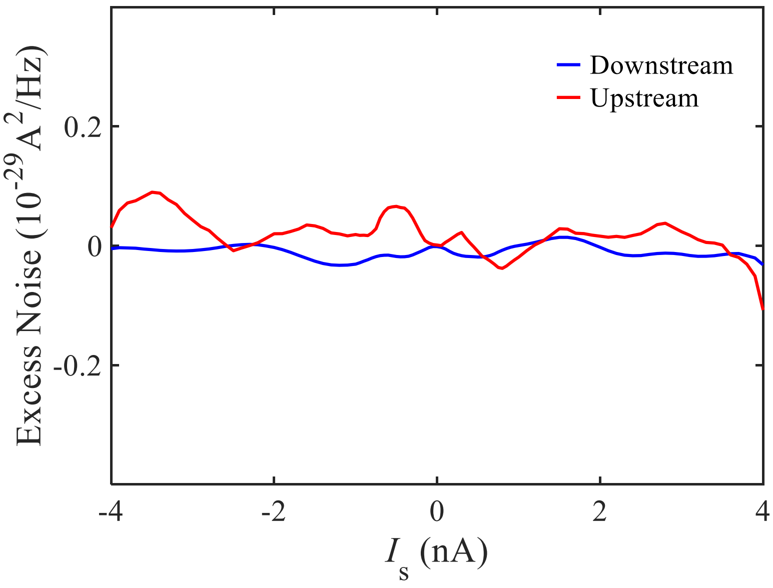

Supplementary Figure 4. Excess noise measurement for a mode of v=1 for the situation of (1,1) and (1,0) in upper and central regions, respectively. Excess noises are negligible at both downstream and upstream.

Supplementary Note 5 –Magnetic field dependent coupling in the lateral distance

Supplementary figure 5 is a schematic diagram of the edge states and LL energies as a function of the lateral direction. Fermi level position should increase while lowering magnetic fields in order to keep a constant filling factor. As depicted in supplementary figure 5 as magnetic field decreases $E_{f1} \rightarrow E_{f2}$, bringing the Fermi Level closer to the 'crossing point' of the LLs, consequently, increasing coupling strength between the edges (the lowest field is restricted, as can be seen from the fan diagram).

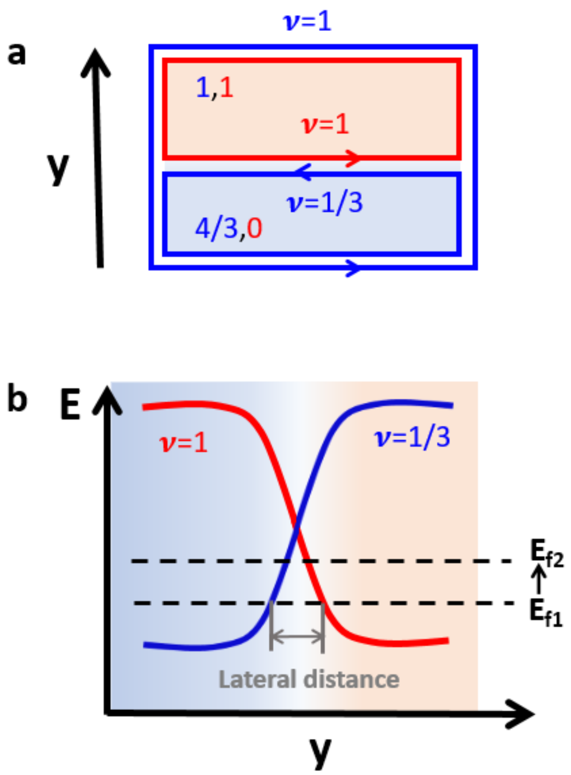

Supplementary Figure 5. A schematic diagram of the edge states and Landau level energies along the y direction. **a.** Schematic diagram of the edge states configuration while the Fermi level lies beneath the intersection point of the LLs. **b**. Complementary scheme of the LLs along the y direction. The ν =1 of the first subband moving around the two regions is dismissed for simplicity. By decreasing magnetic field ($E_{f1} \rightarrow E_{f2}$), bringing the Fermi Level closer to the 'crossing point' of the LLs, where stronger coupling can be obtained.

Supplementary Note 6 – R_{xy} versus center top gate in a DQW with 0.7 nm thick AlAs barrier

Supplementary figure 6 shows the Hall resistance, R_{xy}, measured in a DQW with 0.7 nm thick AlAs barrier, as function of central top-gate when the upper region is at (1,1). The observed R_{xy}=25.8 kΩ and 38.7 kΩ represent that the central region is indeed tuned to (1,0) and (4/3,0), respectively.

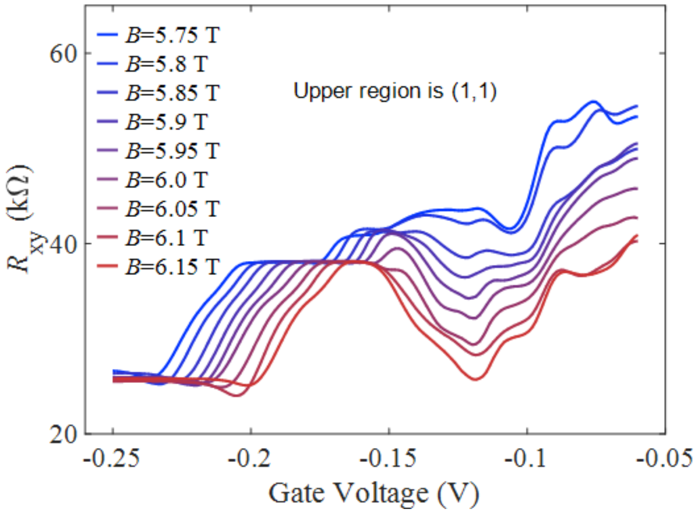

Supplementary Figure 6. Hall resistance in a DQW with 0.7 nm thick AlAs barrier, R_{xy}, vary with top-gate of the central region at different magnetic fields. In this configuration, the filling factor of upper region is at (1,1), thus the observed R_{xy}=25.8 kΩ and 38.7 kΩ represent the central region at fillings (1,0) and (4/3,0), respectively.
